# Simulation and Experimental Study on Roll-Forming Limit of Cup

**DOI:** 10.3390/ma15041279

**Published:** 2022-02-09

**Authors:** Baohong Zhang, Shengpeng Ning, Zeng Wei, Yali Duan, Xubin Li, Yongbiao Yang

**Affiliations:** 1 School of Materials Science and Engineering, North University of China, Taiyuan 030051, China; ningsp0125@163.com (S.N.); weizeng1111@163.com (Z.W.); 2 Engineering Research Center for Magnesium-Base Material Processing Technology, Ministry of Education of China, North University of China, Taiyuan 030051, China; yay20001999@163.com (Y.D.); lixu@nuc.edu.cn (X.L.)

**Keywords:** roll forming, cup, forming limit, DEFORM-3D, damage value

## Abstract

Roll forming can improve the material utilization rate and production efficiency of cups with a curved rotary profile, but there is no basis for the determination of forming limit. The DEFORM-3D software was used to simulate the roll forming of cups. The influence of the billet wall thickness and bottom thickness, coefficient of friction, radius of roller, and the fillet radius of the punch on the forming limit was studied, and the damage value and velocity vector were analyzed. The results showed that the forming limit of the billet’s wall thickness in roll forming for a cup is about 62%. With the increase of the ratio of the formed cup’s wall thickness to the billet’s bottom thickness, the forming limit of wall thickness will be slightly reduced. A larger radius of roller, fillet radius of punch, and friction coefficient between punch and billet and a smaller friction coefficient between roller and billet are good for decreasing the damage value and improving the roll-forming limit. According to the numerical simulation results, the roll-forming limit diagram of cups is established, and the accuracy of the forming limit diagram is verified by experiments.

## 1. Introduction

**Figure 1:**
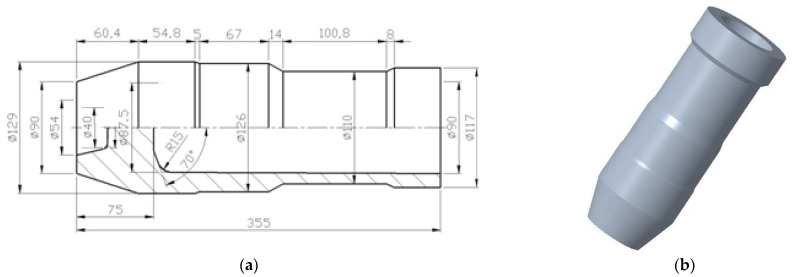
Cup with curved rotary profile: (**a**) schematic diagram; (**b**) 3D model.

Cups with a curved rotary profile (as show in Figure 1) are common parts in industrial production. For example, in projectile bodies, drill pipe joints, etc., the final shape or a certain process in the forming process needs to be processed into a curved rotary profile [1,2,3,4,5]. This kind of part not only has extensive requirements, but also is produced in large quantities. In order to meet the requirements of high mechanical properties and mass production, cups with a curved rotary profile usually use the production process of machining after a cabbage, pierce and draw approach [6,7,8,9,10,11,12]. However, the material utilization rate is relatively low using this kind of production process for a cup with a curved rotary profile, and the longer the length of the part with smaller diameter, the lower the material utilization rate. Aiming at improving the material utilization rate, Xu Hengqiu et al., used the hot-spinning method to form the curved rotary profile of the cup and obtained good profile samples. However, the production efficiency of hot-spinning is low, which cannot meet the requirements of mass production, and it is very difficult to take out the mandrel from the workpiece after hot-spinning [13,14].

**Figure 2:**
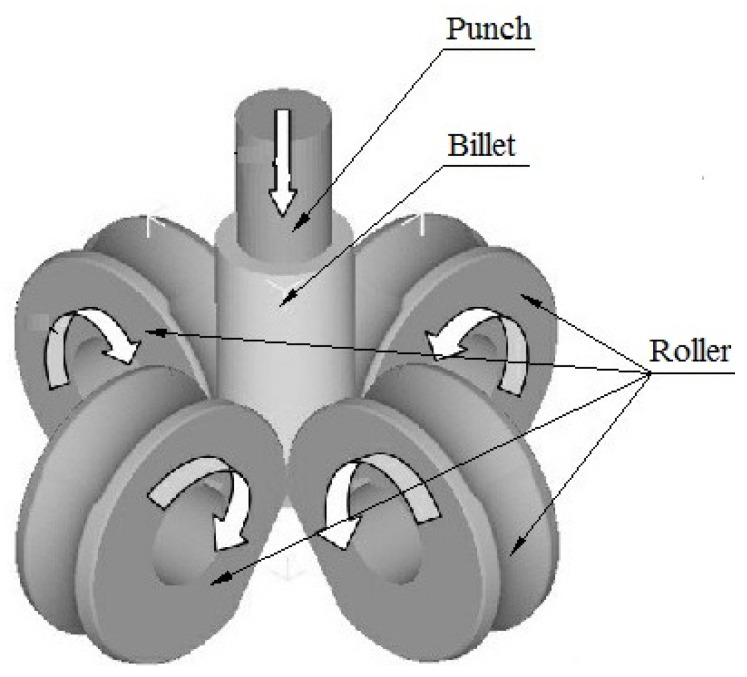
Schematic diagram of roll forming.

**Figure 3:**
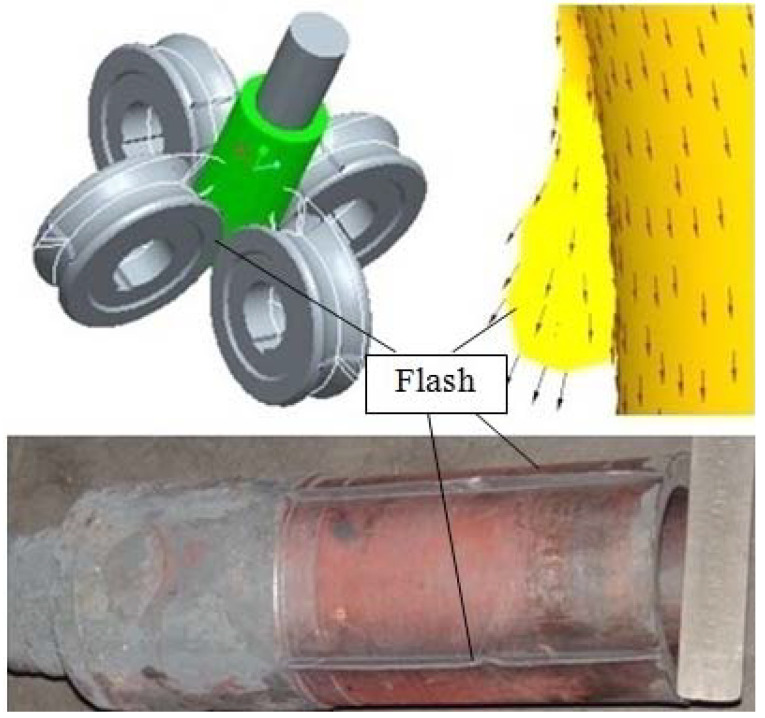
Flash between rollers.

**Figure 4:**
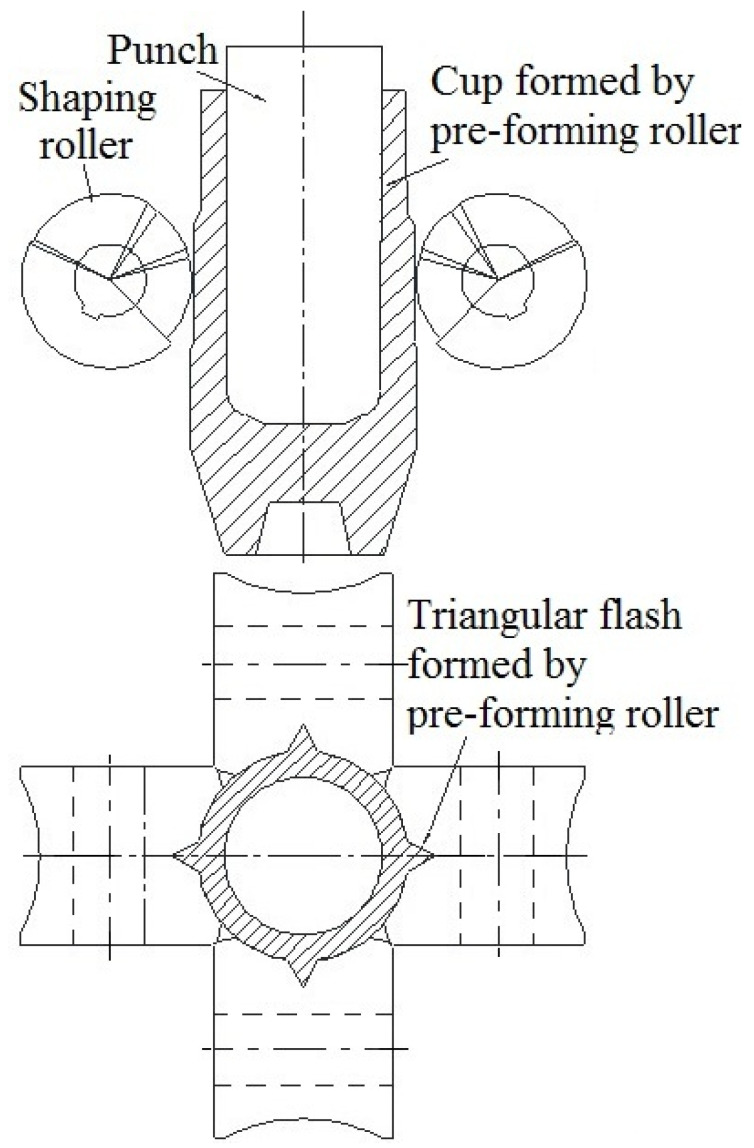
Shaping during roll forming.

For the purpose of both meeting the requirements of mass production and increasing the material utilization rate of a cup with a curved rotary profile, a new technology, roll forming, was proposed on the basis of roll forging and drawing. The theory of roll forming is shown in Figure 2 [15,16,17,18,19,20,21,22,23]. During the process of roll forming, the punch is installed under the movable ram of the hydraulic press. The billet with the cup’s shape is pushed by the downwardly moving punch and makes the pre-forming rollers rotate. The curved rotary profile of the cup is formed by the specific groove shape on the surface of the rollers. During the roll-forming process, a thin flash may be formed at the gap between the rollers as part of the metal may flow into there, which will make it difficult for subsequent machining (as show in Figure 3). A triangular flash groove is designed between the adjacent pre-forming rollers so as to avoid the formation of a thin flash, and then the metal flows into the gap between the rollers and will form a triangular flash. The formed triangular flash can be eliminated by the shaping rollers, which are installed below the pre-forming rollers and rotated at an angle to the forming rollers (as shown in Figure 4). The influence of the triangular flash groove of pre-forming rollers on the quality of roll-formed parts has been studied and the optimized flash groove was put forward [15]. The influence of roller number on roll forming has been studied and the reasonable roller number for roll forming of a cup was suggested [16,17]. The forward slip of roll forming has been studied and the formula for roller design has been proposed [18,19]. The roll-forming process was compared with the traditional process from the aspects of material utilization, productivity, forming force, dies cost and subsequent machining time [20,21].

This method has successfully formed cups with a curved rotary profile [22,23], but there is no basis for determining the roll forming limit, which is one of the most important parameters to determine the forming process [24,25,26,27,28]. During the shaping of roll forming, only the triangular flash formed on the outer surface of the cup in the roll pre-forming is eliminated, and the deformation is small, so only the roll pre-forming limit needs to be studied. In this paper, the roll-forming limit of a cup is studied by means of numerical simulation and experiment. Three or four rollers can be used during the process of roll forming and the method of three rollers is used in this paper.

## 2. Numerical Simulation Model

**Figure 5:**
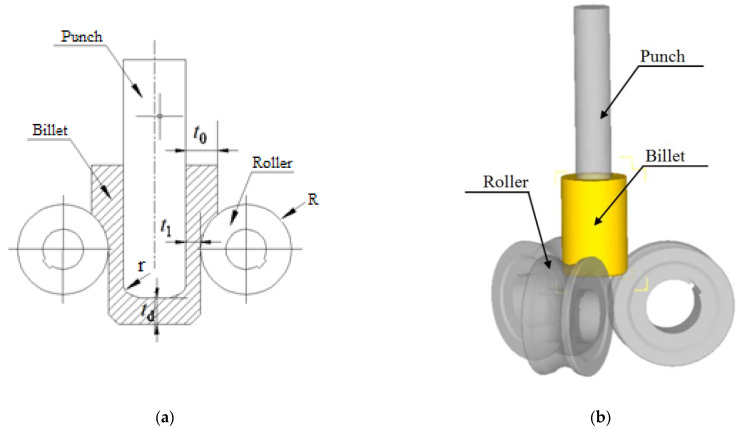
Simulation of roll forming: (**a**) schematic diagram; (**b**) simulation model.

DEFORM-3D finite element software is used for the numerical simulation analysis. In order to study the roll-forming limit efficiently, the roll forming roller is simplified into a groove with the same shape and size. The billet is regarded as a plastic body, the roller and punch are regarded as a rigid body, the material of the billet is pure aluminum 1060 and the material properties according to the supplier are shown in Table 1. The normalized Cockcroft and Latham fracture criterion is adopted [29,30]. According to the actual working conditions of roll forming, the friction coefficient between blank, roller and punch is set to 0.3, the billet’s and the ambient temperature are 20 °C, and the movement speed of the punch is 60 mm/s. The influence of the billet wall thickness and bottom thickness, coefficient of friction, radius of roller, and fillet radius of the punch on the forming limit is studied. The simplified numerical simulation model of roll forming is shown in Figure 5.

## 3. Simulation Results and Analysis

### 3.1. Effect of Billet’s Wall Thickness on Forming Limit

**Figure 6:**
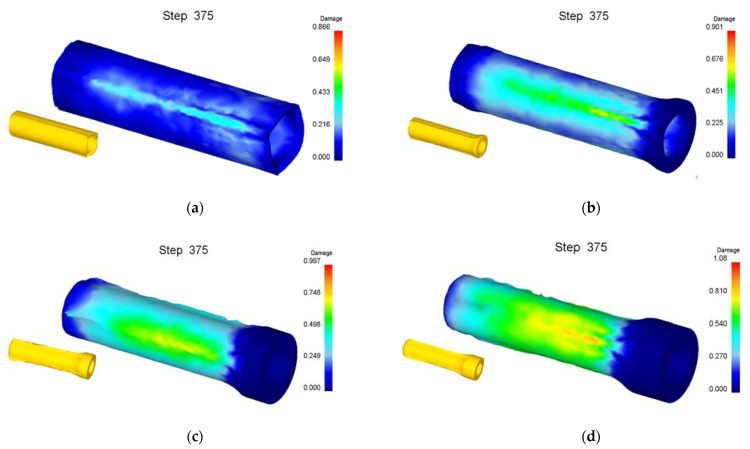
Damage value distribution of roll formed cups by billets with different wall thicknesses of (**a**) 22 mm; (**b**) 26 mm; (**c**) 30 mm; (**d**) 34 mm.

**Figure 7:**
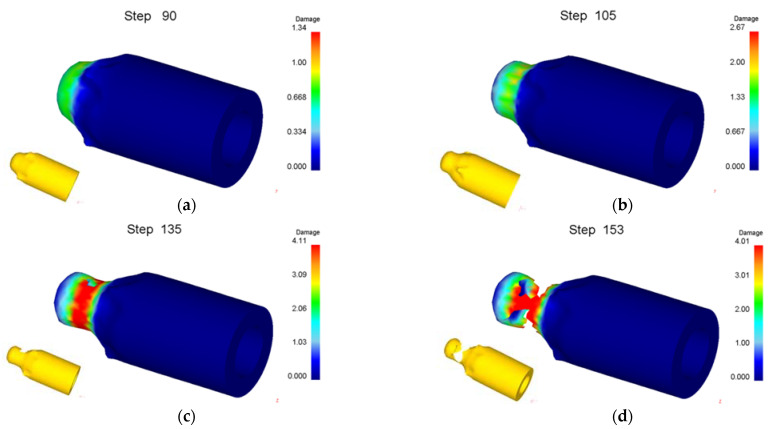
Damage value distribution for forming process of billet with wall thickness of 38 mm. (**a**) Step of 90; (**b**) 105; (**c**) 135; (**d**) 153.

Numerical simulation of roll forming was carried out for five kinds of billets with bottom thickness (*t_d_*) of 40 mm and wall thicknesses (*t*_0_) of 22, 26, 30, 34 and 38 mm, respectively. The wall thickness (*t*_1_) of the billet after forming was 13.5 mm. When the wall thickness of the billet is 22 mm to 34 mm, the damage value distribution of the formed part is shown in Figure 6. It can be seen from Figure 6 that the damage value is small, the shape of the formed part is good and no defects are found. However, with the increase of the billet’s wall thickness, the wall deformation increases, and the force required for the punch to push the bottom of the cup downward increases, resulting in the damage value increasing, the deformation of the fillet at the bottom of the billet’s shape and the enlargement of the fillet. The damage value distribution of the forming process is shown in Figure 7 when the wall thickness of the billet is 38 mm. It can be seen that due to the large deformation of the wall, the bottom of the workpiece shape is greatly deformed when the punch pushes the billet through the roller. With the downward movement of the punch, the workpiece deformation is mainly concentrated at the intersection of the bottom and the wall, resulting in the damage value increasing, the wall being thinned and the bottom and the wall being completely separated finally.

### 3.2. Effect of Billet’s Bottom Thickness on Forming Limit

**Figure 8:**
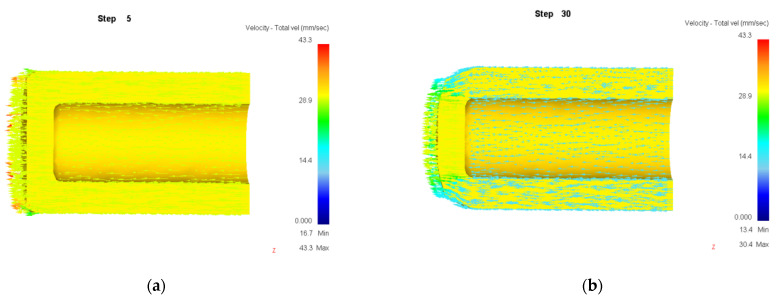

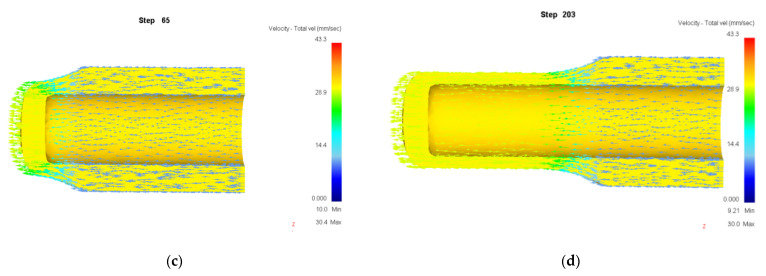
Velocity vector of billet with bottom thickness of 30 mm. (**a**) Step of 5; (**b**) 30; (**c**) 65; (**d**) 203.

Numerical simulation of roll forming was carried out for five kinds of billets with wall thickness (*t*_0_) of 34 mm and bottom thicknesses (*t_d_*) of 10, 20, 30, 40 and 50 mm, respectively. The wall thickness (*t*_1_) of the billet after forming was 13.5 mm. The simulation results show that the roll forming of billets with bottom thicknesses of 30, 40 and 50 mm can be completed smoothly without defects or cracks. The metal flow velocity vector diagram during the forming process with a billet’s bottom thickness of 30 mm is shown in Figure 8. It can be seen that the bottom has sufficient strength to bear the deformation force of the billet wall when the wall thickness is nearly equal to the bottom thickness or the bottom thickness is thick, thus the roll forming can be carried out successfully.

**Figure 9:**
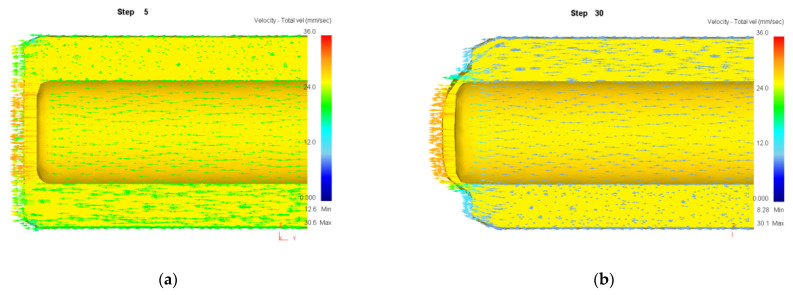

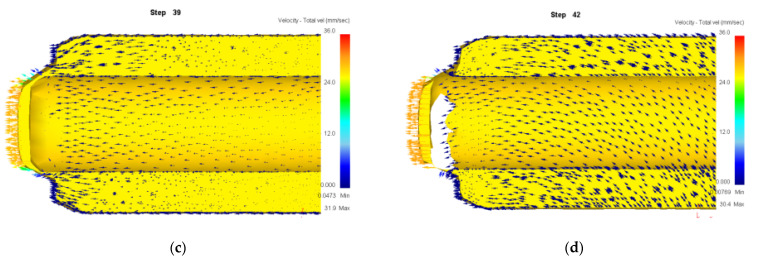
Velocity vector of billet with bottom thickness of 10 mm. (**a**) Step of 5; (**b**) 30; (**c**) 39; (**d**) 42.

When the bottom thickness of the billet is 20 mm, the forming process is nearly same as that in Figure 7, that is, the wall is thinned and fractured. When the bottom thickness of the billet is 10 mm, the metal flow velocity vector diagram during the forming process is shown in Figure 9. It can be seen from Figure 9 that the billet wall only has a small deformation at the intersection with the bottom and the deformation of the bottom is much greater than that of the wall when the punch pushes the bottom of the billet downward. This is caused by the deformation force needed by the billet wall being much greater than that the bottom can take as the bottom of the billet is too thin. The continuous movement of the punch finally resulted in the penetration of the billet bottom by the punch (punching off the billet bottom).

### 3.3. Establishing the Roll-Forming Limit

**Figure 10:**
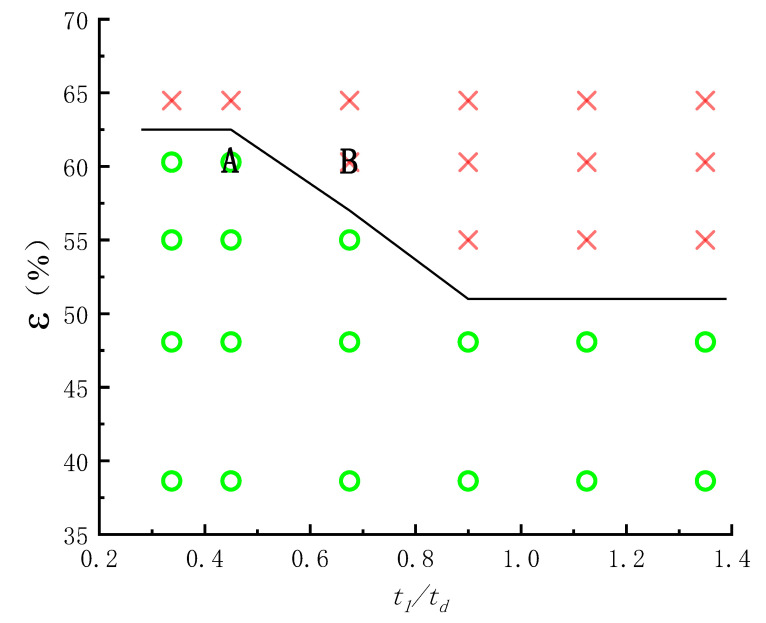
Forming limit diagram of roll-forming for cups.

The above numerical simulation results show that the punch pushes the billet for active motion and the roller is passively driven by the billet during roll forming, but the roll wheel moves actively and the billet moves passively during roll forging, so the factors affecting the roll-forming limit are completely different from roll forging. The forming limit of roll forging depends on whether the billet can be bitten, but the forming limit of roll forming depends on whether the bottom of the workpiece can be penetrated (punched) or whether the wall can be thinned and fractured. When the bottom of the billet is thin and the wall deformation is large, the bottom of the workpiece will be penetrated as shown in Figure 9. When the bottom of the billet is thick and the wall deformation is too large, the problem of wall fracture after being thinned as shown in Figure 7 will occur. When the billet bottom is thick and the wall deformation is moderate, the roll forming can proceed successfully (as shown in Figure 6 and Figure 8). Orthogonal simulation is carried out for different wall thicknesses and the bottom thickness of the billet, and the results are shown in Table 2. The roll-forming limit diagram shown in Figure 10 can be obtained by Table 2. The abscissa in the figure is the ratio of the wall thickness of the formed cup to the bottom thickness of the billet (*t*_1_/*t_d_*), and the ordinate is the deformation of the wall thickness *ε*:

(1) $$\varepsilon =\frac{\left(t_{0}-t_{1}\right)}{t_{0}}\times 100\%$$

where *t*_0_ is the wall thickness of the billet before deformation and *t*_1_ is the wall thickness of the cup after deformation.

It can be seen from the forming limit diagram that when *t*_1_/*t_d_* is less than 0.45, the wall thickness limit deformation that can make roll forming proceed successfully is about 62%. When *t*_1_/*t_d_* is greater than 0.9, the wall thickness limit deformation of roll forming is about 52%. When *t*_1_/*t_d_* is between 0.45 and 0.9, the deformation limit of wall thickness during roll forming decreases linearly with the increase of *t*_1_/*t_d_*.

### 3.4. Effect of Friction Coefficient on Forming Limit

**Figure 11:**
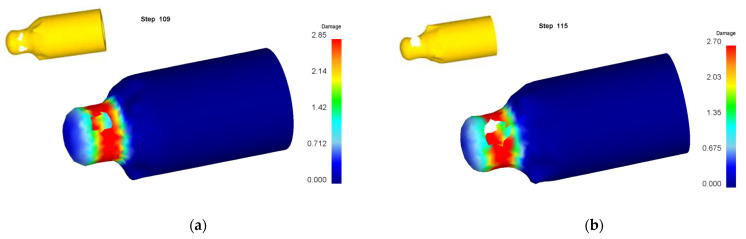

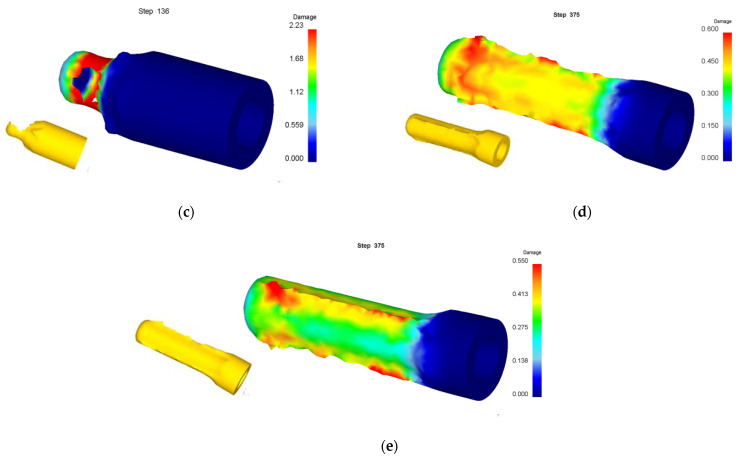
Damage value distribution of different friction coefficients between punch and billet. (**a**) 0.01; (**b**) 0.1; (**c**) 0.3; (**d**) 0.5; (**e**) 0.7.

**Figure 12:**
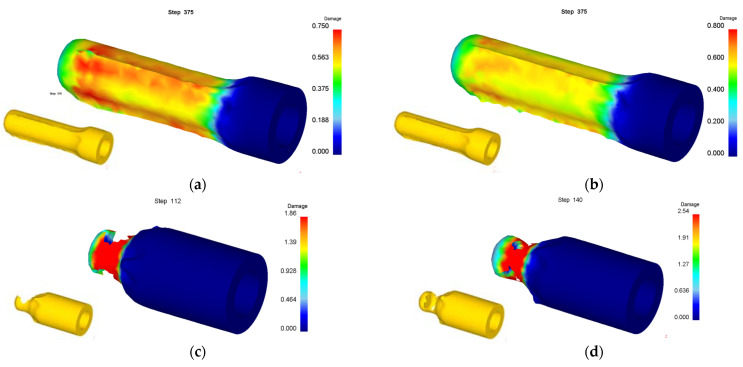

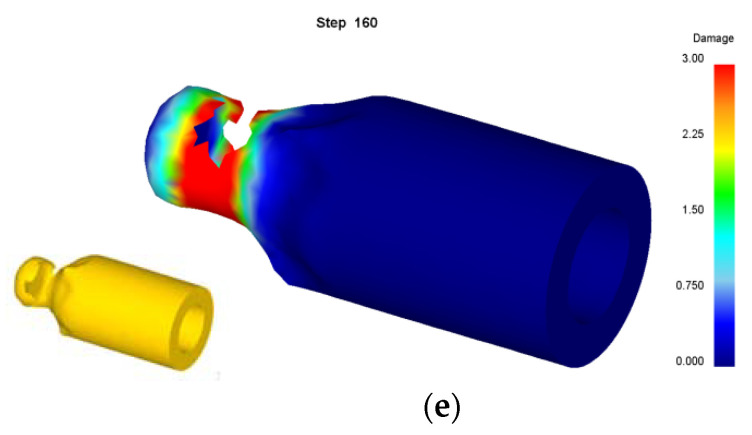
Damage value distribution of different friction coefficient between roller and billet. (**a**) 0.01; (**b**) 0.1; (**c**) 0.3; (**d**) 0.5; (**e**) 0.7.

**Figure 13:**
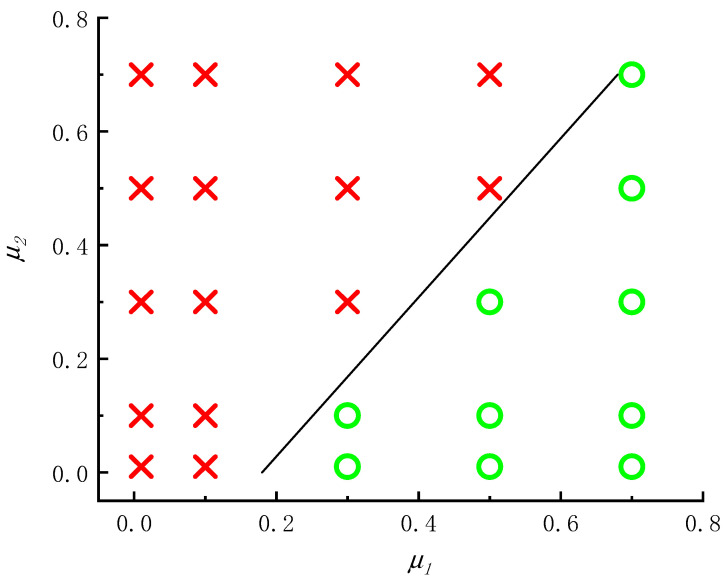
Effect of friction coefficient on forming limit.

The effects of the friction coefficient between punch and billet (*μ*_1_) as well as roller and billet (*μ*_2_) on the forming limit are simulated according to the orthogonal method. The results are shown in Table 3, Figure 11 and Figure 12. According to Table 3, the influence diagram of the friction coefficient between punch and billet and the friction coefficient between roller and billet on the roll-forming limit can be obtained (as shown in Figure 13). It can be seen from Figure 11 to Figure 13 that damage value decreases and the roll-forming limit increases with the increase of the friction coefficient between the punch and the billet, which is mainly due to the increase of the friction coefficient being able to reduce the force of the punch acting on the bottom of the billet. The damage value increases and roll-forming limit decreases with the increasing friction coefficient between billet and roller, which is mainly due to the increase of the friction coefficient leading to the increase of metal flow resistance between rollers.

### 3.5. Effect of Roller Radius on Forming Limit

**Figure 14:**
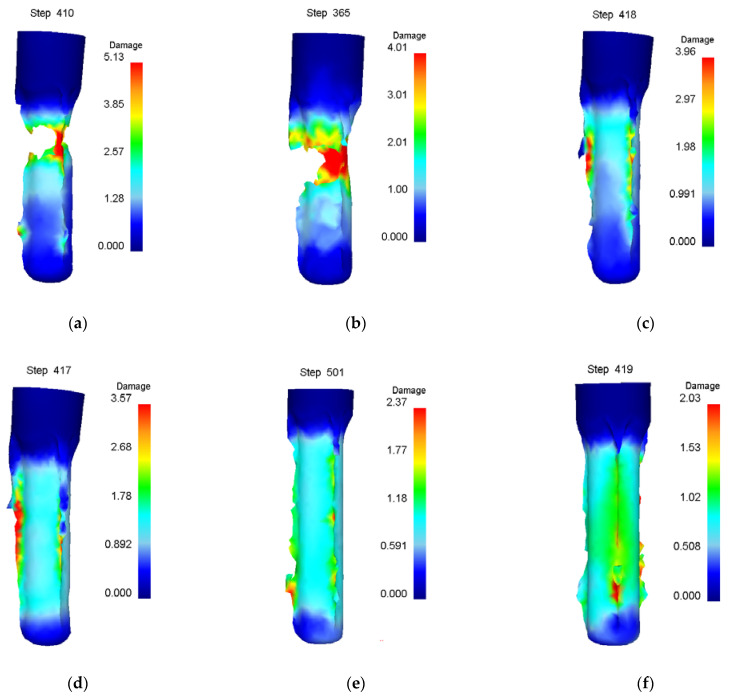
Damage value distribution of different roller radiuses of (**a**) 70 mm; (**b**) 75 mm; (**c**) 80 mm; (**d**) 85 mm; (**e**) 90 mm; (**f**) 95 mm.

Different roller radiuses are simulated and the results are shown in Figure 14 and Table 4. It can be seen from Figure 14, when the roller radius is small and the damage value is large, that the cup’s wall is easily thinned and fractured. With the increasing roller radius, the damage value decreases and the cup’s wall is no longer stretched thin and fractured, and the roll forming can be carried out successfully. This is mainly due to the increasing roller radius being able to increase the contact area between the billet and the roller, which causes the increase of the friction force between the punch and the billet as well as the reduction of the axial tensile force of the billet wall.

### 3.6. Effect of Punch Fillet on Forming Limit

**Figure 15:**
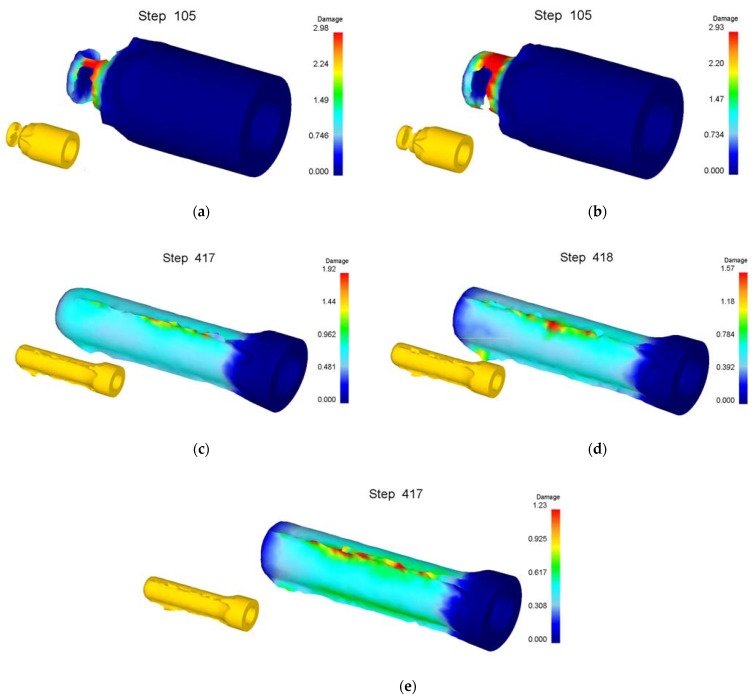
Damage value distribution of different punch fillet radii of (**a**) 5 mm; (**b**) 10 mm; (**c**) 15 mm; (d) 20 mm; (**e**) 30 mm.

Different punch fillets are simulated and the results are shown in Figure 15 and Table 5. It can be seen from Figure 15 that the damage value is large and punching easily occurs at the bottom of the billet when the fillet radius of the punch is small. Increasing the punch fillet radius is conducive to the decreases in damage, the successful progress of roll forming and the improvement of the forming limit. This is mainly due to the small fillet radius of the punch easily producing large stress at the intersection of the cup’s billet wall and the bottom.

## 4. Experimental Verification

**Figure 16:**
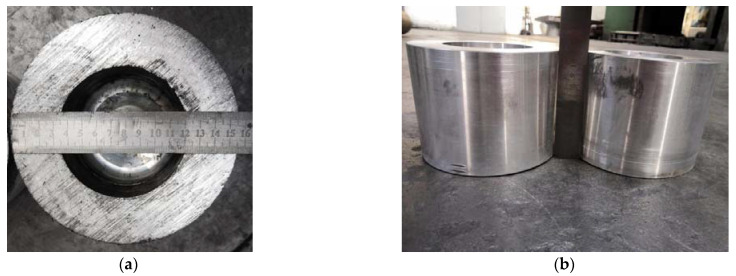
Billets for roll forming: (**a**) top view; (**b**) front view.

**Figure 17:**
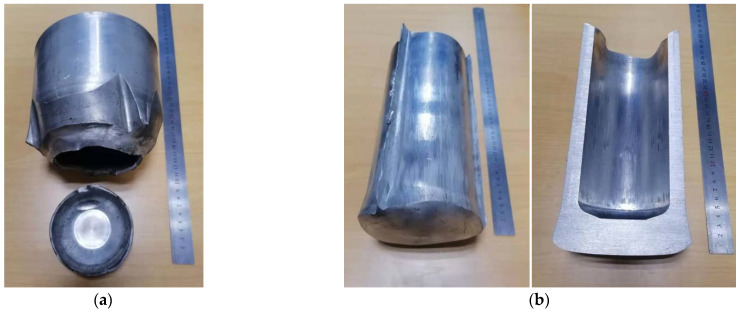
Roll-formed cups: (**a**) bottom thickness of 20 mm; (**b**) 30 mm.

The formable point A and unformed point B near the forming limit (as shown in Figure 10) were experimentally verified on a hydraulic press with 1060 pure aluminum. At the points A and B, the billet wall thickness was 34 mm, and the bottom thicknesses were 30 and 20 mm, respectively. In order to keep the friction coefficient between roller and billet as well as billet and punch the same as 0.3 for the numerical simulation, no lubricant was used in the experiment. The other experimental parameters were consistent with the numerical simulation parameters. The billets and workpiece after roll forming are shown in Figure 16 and Figure 17. It can be seen that the experimental results are consistent with the numerical simulation results, indicating that the roll-forming limit diagram is feasible.

## 5. Conclusions

Based upon experimental and numerical results, the following conclusions are drawn:

- When the bottom of the roll-forming billet is thin and the wall deformation is large, the bottom of the cup is penetrated. When the billet bottom is thick and the wall deformation is too large, the wall of the cup is thinned and fractured. When the billet bottom is thick and the wall deformation is moderate, the roll forming can proceed successfully.
- The forming limit of the billet’s wall thickness in roll forming for a cup is about 62%. With the increase of the ratio of the formed cup’s wall thickness to the billet’s bottom thickness, the forming limit of wall thickness will be slightly reduced.
- The increase of the friction coefficient between punch and billet or the decrease of the friction coefficient between roller and billet can decrease the damage value and improve the roll-forming limit.
- Larger roller radius or punch fillet are good for decreasing damage value and the improvement of the roll-forming limit.
- The reasonable control of process parameters, the bottom thickness of billet, the coefficient between punch and billet as well as billet and punch, the roller radius and the punch fillet, can all decrease the damage value and improve the roll-forming limit of a cup, that is, increase the wall deformation and reduce the forming process.
- The roll-forming limit diagram of a cup was produced and the accuracy of the diagram was verified by experiments, which has important guiding significance for the actual roll-forming of cups.

## Figures and Tables

**Table 1:** Physical and mechanical properties of pure aluminum 1060.

Yield Strength (MPa)	Tensile Strength (MPa)	Elongation (%)	Hardness (HB)	Elastic Modulus (GPa)	Poisson’s Ratio
15	60	22	35	69	0.3

**Table 2:** Orthogonal simulation results of different wall thicknesses and bottom thicknesses of the billet.

No.	*t*_0_ (mm)	*t_d_* (mm)	*μ*_1_	*μ*_2_	*R* (mm)	*r* (mm)	Result
1	22	10	0.3	0.3	95	10	○
2	22	12	0.3	0.3	95	10	○
3	22	15	0.3	0.3	95	10	○
4	22	20	0.3	0.3	95	10	○
5	22	30	0.3	0.3	95	10	○
6	22	40	0.3	0.3	95	10	○
7	26	10	0.3	0.3	95	10	○
8	26	12	0.3	0.3	95	10	○
9	26	15	0.3	0.3	95	10	○
10	26	20	0.3	0.3	95	10	○
11	26	30	0.3	0.3	95	10	○
12	26	40	0.3	0.3	95	10	○
13	30	10	0.3	0.3	95	10	×
14	30	12	0.3	0.3	95	10	×
15	30	15	0.3	0.3	95	10	×
16	30	20	0.3	0.3	95	10	○
17	30	30	0.3	0.3	95	10	○
18	30	40	0.3	0.3	95	10	○
19	34	10	0.3	0.3	95	10	×
20	34	12	0.3	0.3	95	10	×
21	34	15	0.3	0.3	95	10	×
22	34	20	0.3	0.3	95	10	×
23	34	30	0.3	0.3	95	10	○
24	34	40	0.3	0.3	95	10	○
25	38	10	0.3	0.3	95	10	×
26	38	12	0.3	0.3	95	10	×
27	38	15	0.3	0.3	95	10	×
28	38	20	0.3	0.3	95	10	×
29	38	30	0.3	0.3	95	10	×
30	38	40	0.3	0.3	95	10	×

*t*_0_: wall thickness of billet; *t_d_*_:_ bottom thickness of billet; *μ*_1_: friction coefficient between punch and billet; *μ*_2_: friction coefficient between roller and billet; *R:* radius of roller; r: radius of punch fillet; o: roll forming is successful; x: roll forming is unsuccessful.

**Table 3:** Simulation results of different friction coefficients.

No.	*t*_0_ (mm)	*t_d_* (mm)	*μ*_1_	*μ*_2_	*R* (mm)	*r* (mm)	Result
1	38	40	0.01	0.01	95	10	×
2	38	40	0.1	0.01	95	10	×
3	38	40	0.3	0.01	95	10	○
4	38	40	0.5	0.01	95	10	○
5	38	40	0.7	0.01	95	10	○
6	38	40	0.01	0.1	95	10	×
7	38	40	0.1	0.1	95	10	×
8	38	40	0.3	0.1	95	10	○
9	38	40	0.5	0.1	95	10	○
10	38	40	0.7	0.1	95	10	○
11	38	40	0.01	0.3	95	10	×
12	38	40	0.1	0.3	95	10	×
13	38	40	0.3	0.3	95	10	×
14	38	40	0.5	0.3	95	10	○
15	38	40	0.7	0.3	95	10	○
16	38	40	0.01	0.5	95	10	×
17	38	40	0.1	0.5	95	10	×
18	38	40	0.3	0.5	95	10	×
19	38	40	0.5	0.5	95	10	×
20	38	40	0.7	0.5	95	10	○
21	38	40	0.01	0.7	95	10	×
22	38	40	0.1	0.7	95	10	×
23	38	40	0.3	0.7	95	10	×
24	38	40	0.5	0.7	95	10	×
25	38	40	0.7	0.7	95	10	○

**Table 4:** Simulation results of different roller radiuses.

No.	*t*_0_ (mm)	*t_d_* (mm)	*μ*_1_	*μ*_2_	*R* (mm)	*r* (mm)	Result
1	38	40	0.3	0.3	70	10	×
2	38	40	0.3	0.3	75	10	×
3	38	40	0.3	0.3	80	10	○
4	38	40	0.3	0.3	85	10	○
5	38	40	0.3	0.3	90	10	○
6	38	40	0.3	0.3	95	10	○

**Table 5:** Simulation results of different punch fillet radii.

No.	*t*_0_ (mm)	*t_d_* (mm)	*μ*_1_	*μ*_2_	*R* (mm)	*r* (mm)	Result
1	38	40	0.3	0.3	95	5	×
2	38	40	0.3	0.3	95	10	×
3	38	40	0.3	0.3	95	15	○
4	38	40	0.3	0.3	95	20	○
5	38	40	0.3	0.3	95	30	○

## Data Availability

The data supporting reported results are available on request from the corresponding author.

## Abbreviations

Abbreviations, acronyms, and symbols

*t*_0_	Wall thickness of billet
*t_d_*	Bottom thickness of billet
*t*_1_	Wall thickness of deformed cup
*μ*_1_	Friction coefficient between punch and billet
*μ*_2_	Friction coefficient between roller and billet
*R*	Radius of roller
*r*	Radius of punch fillet
o	Roll forming is successful
x	Roll forming is unsuccessful

## References

[1] Zhu X.H., Zhang Z. (2017). Design of an ultra-high torque double shoulder drill-pipe tool joint for extended reach wells. Nat. Gas Ind. B.

[2] Dong L.L., Zhu X.H., Yang D.S. (2019). Study on mechanical behaviors of double shoulder drill pipe joint thread. Petroleum.

[3] Liu Y.M., Xiong Z.M., Lu H., Hu J.F., Rong X.L. (2018). Study on overload characteristics of rigid projectile body high-speed impact metal network. J. Ord. Equip. Eng..

[4] Li C.L., Wang Y.S., Zhang Z.B., Zhu L.L., Xiao J. (2018). Summary of Swedish 84mm Gustav recoilless ammunition technology. J. Ord. Equip. Eng..

[5] Zhou Z.B., Zhang B., Guo S.F., Gu H.P., Yuan B.H. (2021). Experimental study on ballistic deflection of different warheads penetrating steel plate. J. Ord. Equip. Eng..

[6] Li L.Q., Li X.H., Gao B.B. (2013). Summary of key manufacturing technologies of highly efficient warhead projectile abroad. Def. Manuf. Technol..

[7] Kang B.S., Lee J.H., Kim S.H. (1997). Development of a methodology to form netshape nosing shells by the backwards tracing scheme of the rigid-plastic FEM. Intl. J. Mach. Tools Manuf..

[8] Zhu J. (1997). A new approach to preform design in shell nosing. J. Mater. Process. Technol..

[9] Hwang S.M., Kobayashi S. (1987). Preform Design in Shell Nosing at Elevated Temperatures. Intl. J. Mach. Tools Manuf..

[10] Zhang B.H., Zhang Z.M., Zhang X. (2004). Numerical simulation of warm drawing of cup. J. Plast. Eng..

[11] Zhang Z.M., Huang S.D., Pang D., Song F., Zhao Z.X., Zhao Z.D. (2013). Research and development of database for hot drawing process of projectile. J. Netshape Form. Eng..

[12] Jiang C.M., Liu H.Y., Fu W., Wang J.Y., Fang X.L., Qiu Z.B., Dong Z.X. (2016). Research on the heat-sealing forming process of the projectile blank. J. Netshape Form. Eng..

[13] Xu H.Q., Zhang R., Wang D.L., Huang T., Guo L., Zheng H.W., Gao W.L. (2014). Research on technology of hot spinning forming of parts of 60Si2Mn steel with curvilinear. New Technol. New Process..

[14] Zhan M., Yang H., Guo J., Wang X.X. (2015). Review on hot spinning for difficult-to-deform lightweight metals. Trans. Nonferrous Met. Soc. China.

[15] Hu B., Zhang B.H., Wei C.Y., Zhang Z.M. (2020). Influence of opening angle of roller flash groove on cylinder quality of roller forming. J. Plast. Eng..

[16] Sun H., Zhang B.H., Li X.B., Zhang Z.M., Duan Z.Y. (2019). Influence of roller number on quality of cylinder parts in roll extrusion forming. Form. Stamp. Technol..

[17] Zhang B.H., Hu B., Wei C.Y., Zhao X. (2021). Roll Forming of cup with curved rotary profile. Proceedings of the 13th International Conference on the Technology of Plasticity, Virtual Event, 25–30 July 2021.

[18] Zhang B.H., Hu B., Zhang Z.M., Zhao X. (2020). Influence of process parameters on the forward slip of cup during roll forming. J. Phys. Conf. Ser..

[19] Zhang B.H., Hu B., Zhang Z.M., Zhao X. (2020). Study on the forward slip of cup with variable wall thickness during roll forming. J. Phys. Conf. Ser..

[20] Zhang B.H., Hu B., Wei C.Y., Zhao X., Zhang Z.M. (2021). Roll forming of projectile body with curved rotary profile. J. Phys. Conf. Ser..

[21] Zhang B.H., Wei C.Y., Hu B., Zhao X. (2020). Roll-drawing of cup with thick wall. Procedia Manuf..

[22] Zhao X., Zhang Z.M., Zhang B.H. (2013). Finite element simulation on rolling-extrusion forming of variable wall thickness cylinder parts. J. Chem. Pharm. Res..

[23] Zhao X., Zhang Z.M., Zhang B.H. (2014). Research on rolling-extrusion forming of variable wall thickness cylinder parts. Mater. Res. Innov..

[24] Yu Q., Liang J., Li Q., Li C. (2021). Development of Measurement Equipment and Experimental and Numerical Simulation Studies for Warm Forming Limits of High-Strength Steel. Materials.

[25] Pandre S., Morchhale A., Kotkunde N., Singh S.K. (2020). Influence of processing temperature on formability of thin-rolled DP590 steel sheet. Mater. Manuf. Process..

[26] Chen J., Gong P., Yang L. (2020). Forming limit evaluation for AA5182 aluminum alloy at warm temperatures based on M–K model. J. Mater. Eng. Perform..

[27] Li J., Xie X., Yang G., Du C., Yang L. (2017). Forming limit diagram determination using Digital Image Correlation: A Review. Conference Proceedings of the Society for Experimental Mechanics Series.

[28] Behrens B.A., Uhe J., Wester H., Stockburger E. Hot forming limit curves for numerical press hardening simulation of AISI 420C. Proceedings of the 29th International Conference on Metallurgy and Materials (Metal 2020) Conference Proceedings.

[29] Saanouni K. (2008). On the numerical prediction of the ductile fracture in metal forming. Eng. Fract. Mech..

[30] Yu S., Feng W.M., Wang R. (2010). Research on ductile fracture criterion in plastic deformation processes. Form. Stamp. Technol..

